# Supplementary material for: A qualitative study of positive psychological experiences and helpful coping behaviours among young people and older adults in the UK during the COVID-19 pandemic
Source: PLoS One. 2023 Jan 23;18(1):e0279205. doi: 10.1371/journal.pone.0279205 (PMC9870142; doi:10.1371/journal.pone.0279205)
Supplement: S2 Fig — (DOCX) [file pone.0279205.s002.docx]

**S2 Figure 2:** Interview Guide Example Questions and Prompts Pertinent to the Research Question

| - Have you experienced any impact on positive emotions? Prompts:   - How deeply you can engage with what you are doing   - Sense of meaning/ purpose   - Relationships with others   - How well you are managing and feelings of control over your situation - Have you been doing/ planning anything to help with this? Prompts: - Connecting with family or friends/ work colleagues online? - Online groups? - Hobbies/ Reading - Exercise at home - Volunteering - Other engagement |
| --- |
