# Supplementary material for: A qualitative study of positive psychological experiences and helpful coping behaviours among young people and older adults in the UK during the COVID-19 pandemic
Source: PLoS One. 2023 Jan 23;18(1):e0279205. doi: 10.1371/journal.pone.0279205 (PMC9870142; doi:10.1371/journal.pone.0279205)
Supplement: S1 Table — (DOCX) [file pone.0279205.s003.docx]

**S1 Table 1:** Timeline of COVID-19 Restrictions in UK Between 2020 and 2021 and Proportion of Study Interviews Conducted in Each Month

| **Time Period / Percentage of Interviews Conducted^3^** | **Covid-19 Restrictions** |
| --- | --- |
| Mar – Apr 2020  *No interviews conducted* | 20 Mar 2020: Schools and colleges close. Entertainment, hospitality and indoor leisure premises close.  22 Mar 2020: Announcement of new shielding measures for people identified as highest risk of severe illness from COVID-19 – these people (which included many older adults) were advised to ‘shield’ at home at all times for a period of at least 12 weeks.  23 Mar 2020: First national lockdown begins. People ordered to “stay at home”. Legal exemptions for leaving home include essential shopping, going to work, medical reasons, exercising once per day. |
| May 2020  *16% of interviews conducted* | 13 May 2020: First step in easing lockdown measures starts. This includes: (1) those who cannot work from home encouraged to return to work but avoid public transport; (2) unlimited outdoor exercise in public spaces is permitted; (3) people allowed to meet someone from one other household outside. |
| Jun 2020  *12% of interviews conducted* | 1 Jun 2020: Second step in easing lockdown measures starts. This includes: (1) people who have been ‘shielding’ are allowed to spend time outdoors with members of their household; (2) outdoor gatherings of six people and indoor gatherings of two people from different households permitted; (3) schools begin to reopen.  15 Jun 2020: Third step in easing lockdown measures starts. This includes: (1) non-essential shops reopen in England; (2) secondary schools begin to offer face-to-face support for a quarter of the year 10 and 12 cohort at any one time, to supplement remote education. |
| Jul 2020  *13% of interviews conducted* | 4 Jul 2020: Fourth step in easing lockdown measures starts. This includes: certain businesses including hospitality (e.g., restaurants, pubs, cafés), some leisure facilities and attractions (e.g., museums, cinemas, libraries, community centres, outdoor gyms) can reopen. Local lockdowns introduced for areas with high rates of Covid-19 infection. |
| Aug 2020  *21% of interviews conducted* | 1 Aug 2020: ‘Shielding’ guidance paused which means that those who were previously shielding can go to work as long as the workplace is COVID-19-secure, but should carry on working from home wherever possible; can go outside but should still try to keep overall social interactions low; can visit businesses with social distancing.  15 Aug 2020: Reopening of a number of establishments within the culture, sport, leisure and business sectors including indoor theatres, bowling alleys. |
| Sep 2020  *16% of interviews conducted* | 14 Sep 2020: Socialising is limited to groups of six people.  24 Sep 2020: New social distancing restrictions come into place including: (1) advise to work from home where possible; (2) 10pm curfew for hospitality venues introduced. |
| Oct 2020  *10% of interviews conducted* | 14 Oct 2020: Three tiered system of local COVID-19 Alert Levels introduced where different regions of the country will live under different restrictions depending on local infection rates. |
| Nov 2020  *7% of interviews conducted* | 5 Nov 2020: Second national lockdown begins. This includes: (1) people must ‘stay at home’ except for work or volunteering that cannot be done from home or essential activities; (2) people over the age of 70 or ‘with more general underlying health conditions’ are advised to minimise contact with others and to stay at home as much as possible; (3) schools, colleges and universities will remain open but with further guidelines. |
| Dec 2020  *No interviews conducted* | 2 Dec 2020: Second national lockdown ends and England moves back to a tiered system of local restrictions. Updated guidance for those who are shielding reflects the move back to a tiered system of local restrictions.  20 Dec 2020: The new tier 4 alert level: ‘stay at home’ comes into place across parts of England where residents within Tier 4 areas are required to ‘stay at home’ by law except for exercise or other legally permitted reasons, and with exemptions for certain work, education, childcare purposes. Reintroduction of shielding advice in Tier 4 areas – clinically extremely vulnerable individuals are advised to stay at home unless for exercise or medical appointments and not to attend work, even if unable to work from home |
| Jan 2021  *4% of interviews conducted* | 6 Jan 2021: England enters third national lockdown. This includes: (1) stay-at-home order except for work, or to provide voluntary or charitable services that cannot reasonably be done from home; (2) closure of schools to most pupils and all primary schools, secondary schools and colleges will move to remote learning; (3) clinically extremely vulnerable are advised to begin shielding again.  27 Jan 2021: Prime Minister confirmed that lockdown measures will stay in place until at least 8 March 2021. |

^1^This list is focused on COVID-19 measures specific to young people and older adults but is not exhaustive. Restrictions were similar across England, Scotland, Wales and Northern Ireland; however dates and precise details may vary.

^2^Brown & Kirk-Wade (2021); Dunn et al. (2020)

^3^Of the total number 68 interviews conducted for this study
