# Supplementary material for: A qualitative study of positive psychological experiences and helpful coping behaviours among young people and older adults in the UK during the COVID-19 pandemic
Source: PLoS One. 2023 Jan 23;18(1):e0279205. doi: 10.1371/journal.pone.0279205 (PMC9870142; doi:10.1371/journal.pone.0279205)
Supplement: S2 Table — (DOCX) [file pone.0279205.s004.docx]

**S2 Table 2:** Minimal Data Set

| **Theme** | **Subtheme** | **Quote example (age 13-24)** | **Quote example (age 70-93)** |
| --- | --- | --- | --- |
| **Theme 1: Engagement in self-fulfilling activities** | **Opportunity for leisure and exploration of new skills** | “I’ve took up making things out of resin, like jewellery and stuff. So, that’s been quite helpful, to just sit and concentrate on one thing.”  “I have been able to put my time into finding a couple of new interests, like the sound design and with rap, actually, that I’ve got into. Which has been quite cool.”  “I’ve started reading much more, because the problem was I got so bored I ended up on my laptop. And then, because I was spending all day on my laptop with classes, when it came to the end of the school day I didn’t want to really go on my screen anymore. So, it was really nice to be able to read my book.”  *‘I think I’ve improved on my music a lot more because I had more time to practice’*  *‘I haven’t needed to worry about doing a job so I have money next month. So in a way, I’ve been able to relax a bit which has been really nice and not focused everything on my work. So that is where I’ve been able to dedicate time to help with anything outside and I actually produce video content for those and learn new skills when it comes to software...’* | “I’ve now started to ride a proper bike as well. I live in a Close, so we don’t get any through traffic and I can cycle around that Close and I do a few laps. But I haven’t ridden a bike for 60 years”  “I focus on mainly two things. One is getting things done in the garden and the other one is doing some serious reading. [The pandemic] created a situation where I get on with those without feeling guilty. I was in the habit of putting into my calendar, once a month I’d put in a reading morning and also a reading afternoon to actually give myself permission to do nothing that morning except read. I don’t need to do that now.”  *‘I’ve always loved reading so I’ve been able to get on with as much reading as I want. …to actually just sit for a couple of hours reading and not feel guilty.* |
|  | **Opportunity to organise affairs** | “I suppose kind of a sense of satisfaction that it’s giving me the space to finally get around to fixing certain aspects of my life. Sorting out routines and getting finally around to doing the exercise and stuff, meditating more the sort of general wellness personal admin stuff. I’m glad that that’s happened, I wish it could have happened in circumstances other than this.” | “…we sat down and made up a list of projects, if you like, of things that we’ve been planning to do, things that need to be done.”  “I was thinking of trying to sort out all my papers, so that when I die, they can find things. I’ve got plenty of time to do that. So, I’m thinking about that, which is probably no bad thing.”  *It (the pandemic)’s given me the focus to get into the garden and get on with that because for seven years I was at best maintaining it. Last year I made a big effort to get on top of it. …and the lockdown has enabled me to get on with that.* |
| **Theme 2: Increased sense of social cohesion** | **Heightened compassion and connectedness** | “I guess I’m a bit more careful about… Not careful. A bit more watchful of my friends, just to see how they are, a bit more. The importance of just checking up on them to see if they have any concerns.”  *‘There are some people I haven’t necessarily missed not seeing them. ... I’ve connected with the people I’ve needed to. So, in a way my network it’s become clearer and seems closer.’*  *‘it (the pandemic) was like a friends filter’* | “I show more compassion to people. I appreciate the kindness that I’ve been offered… People are supportive, and I will remember that. I’ll remember the kindnesses I’ve been shown.”  “it’s deepened [my relationships], because it makes you think about what really matters, as I said before, and what really matters is your interaction with the people around you. Your relationships with the people around you, and the things you share with the people that you don’t necessarily know.”  *‘So, it’s going to be worth making more of in the future, making more effort about in the future. Family, it makes me realise that I do really love them and miss them.* |
|  | **Greater sense of community** | “during the initial bits of lockdown, there was quite this sense of solidarity, wasn’t it? This general feeling of, do you know what, this sucks, but we’re all in this together. We just have to get on with it, right? That was really nice, I think.”  “I’m more aware of what’s going on in my community. I’m part of more community groups now. I see all the updates from the food bank and things like that…”  “I think another positive of the pandemic is that it’s brought more people together which sounds cringey but what I mean by that is definitely with our neighbours we’ve become more close because we’re at home all the time. So a lot of the time if we bake something, like a cake or something, we would give some to our neighbours and then they would do the same for us.” | “Early on, we set up a village group, well group’s not the right word, but a phone number, took a flyer round to everybody’s house with a phone number, that people who were in lockdown could phone so we’d go and get prescriptions, we’d get essential food, that sort of thing. That’s really good, as well.”  “I [previously] thought the idea of having a picnic out of your front porch, [was] a ghastly idea, sitting on your own with a cup of tea. But, because there was enough space, people could sit in their front gardens with the wall in between them, but still chat to people passing by. So, there was a huge sense of community, but a very safe sense of community, which is brilliant.”  *‘We relate a bit more to the local neighbours. … They all come out and clap every Thursday night at 8 o’clock… So, chatting more to the neighbours is probably the main positive.* |
| **Theme 3: Greater personal growth** | **New outlook on gratitude** | “I will probably try not to take for granted going out and going to school and hugging people definitely. Yes, I think I’ll try not to take that for granted”  “in a way it helped, because before I was taking everything for granted. And when I would see my friends I wasn’t as excited, because I knew that I might see them the next day. And then there was one day where I was finally able to go and see my friend, just before I went back to school. And I don’t think I’ve ever been more excited to go out and just see her, even though we were socially distanced. Just be able to be there in person was so nice. And in a way it was a nice change, even to be excited to go back to school.”  “I guess it’s really made me value spending time with people, as I’m sure it has with everyone. Even though I’m not very extroverted, I do like to spend some time with the people that I love, and it made me realise how special that is and how I really need to spend more time with these people.”  *I think one of the downsides of the pandemic was meeting new people wasn't really possible. And one thing I was really grateful for was that I am in a happy relationship with someone for the past two years. But some friends of mine who were single were struggling with this idea of trying to meet new people to date. …that made me very grateful that I do have a partner and three different people in my household I can rely on.* | “I feel very fortunate that [husband] and I have not suffered the terrible dilemmas that people are experiencing because they’re not as fortunate. We’re retired so we have our pensions, so we’re not having to worry about losing our jobs, losing our income or finding a way in which to work in order to be able to earn some money when there can’t be any contact between people… I just felt so sorry for those people and grateful that I’m not in that situation.”  *We’re retired so we have our pensions, so we’re not having to worry about losing our jobs, losing our income or finding a way in which to work in order to be able to earn some money when there can’t be any contact between people. So I feel very fortunate that we’re not faced with that problem*.  “I think that hopefully, if everything works out all right, one should appreciate normality more than we have done. You take things for granted. You take for granted the idea that you can get in the car and perhaps drive to the seaside and have a nice walk there. I think one’s likely to be able to appreciate these things and also obviously the actual contact of going to the groups and going to church. I think hopefully we shall all feel much more appreciative of the normality of things than we have in the past.” |
|  | **Increased feelings of resilience** | “Meditation, having that self-care, very useful just to build resilience. So, when bad stuff happens, I was very glad that I had that as a backup and that I’d been working on it.”  “I feel like I also increased my ability to look after myself mentally and physically. Like I said with the structure and the regime and things like with being able to make a structure that I can stick to. I stuck pretty religiously to it”  *‘I now know how important it is to have good mental health, constantly. So, that if anything like this were to happen again, I would be prepared.’* | “I’ve surprised myself really. That I’m not… That’s what I mean about this fear thing. I’m not anxious in the way that I have in the past. So that’s been very good.”  “I think I’m stronger than I thought I was, and that I can do things, possibly, I didn’t think I would be able to do, and I’ve coped better than I thought I’d be able to cope.”  *I'm probably proud of myself, how I've dealt with it (a heart attack) ... Sort of managing my own symptoms, managing my own time, and in a way, being strong enough to say, I'm going to go to bed today. Rather than doing the socially acceptable thing of pushing myself... So, I'm quite proud of myself that I've been able to do that* |
| **Theme 4: Use of problem-focused strategies to manage pandemic-related stressors** | **Managing intake of pandemic-related news** | “…not reading the news because whenever I read the news it’s always just bad things. It always makes me worry, so just staying away from that helped… We watched the government briefings and stuff like that which I never really watched any before but then obviously around the time of lockdown starting I did because I just wanted to know what was going on. But then I tried to not watch it as much because it just made me worried and stuff.”  *‘whenever I read the news it’s always just bad things. It always makes me worry, so just staying away from that helped as well.’* | “I felt anger. Anger. I feel cross sometimes, really cross. I tell you one thing, I won’t listen to the news now. I switch the news off as soon as it comes on, I refuse to listen to the news, and when people insist on telling me something that’s been on, I feel annoyed about it. I think the news is not helping, I don’t think it helps. I think people who listen to the news are getting depressed, so I thought, right, I won’t listen to the news, so I don’t any more. I don’t read any newspapers. I was somebody who always kept up with the news and things like that. I won’t do that, because I knew that was having an impact on me”  *‘cutting down on news bulletins but nevertheless, making sure that one is in touch with what is happening, is probably advantageous’* |
|  | **Adopting a new routine** | “My mum and I have started this thing where we do 8,000 steps every day, which is about an hour’s walk. Before lockdown I really wasn’t doing that, and it’s pretty helpful, you know? It gave me a chance to chat to her about my day, tell her what’s going on, get away from the house for a bit, and spend some quality time. Obviously, exercise has a known benefit, so that definitely helped… With my schoolwork, I made a timetable just so I felt a bit more under control.”  “I think the important things to me over lockdown have been actually to give myself a bit of a schedule and wake up by around 7:00, 7:30 in the weekdays than actually have a lie-in on the weekdays. And to be sure that I go for a run and get some exercise and go for a walk somewhere nice. We go somewhere at least once a week and also just spend time cooking and gardening and appreciating all the smaller things that sometimes get so rushed. So I have been really nicely kind of having a shift and have a bit more of a relaxed life in away.” | *I do make myself behave as if, yes, I’m going out to do something. Even if it’s only to go for a walk, or do the garden, that is my thing and I’m going to do it properly. … and I feel, therefore, positive that I have done things, and achieved things* |
| **Theme 5: Use of strategies to regulate thoughts and emotions** | **Engaging with arts and digital mental health apps** | “I was able to continue my ballet, sort of. And I was also able to continue my singing, which I was very glad of. But I did that over FaceTime. And I was able to continue doing my grades. And now I’m going to be also able to complete my grade online as well. So, I was very lucky to be able to do that.”  “I now have a mood tracking app on my phone, which I have downloaded so that I can track my mood and see what it affects. It’s a very useful app. It can show you different advice, and if you’ve said that you’re constantly tired for so many days, or if you feel a certain way, it can say I understand you’re having sleep trouble, and it will give you an article about what you can do… So that’s something I’ve taken up which I think I’m going to keep doing, because I think it’s very helpful.” | “The radio is amazing, the radio’s fantastic, I listen to that. I’ve always listened to the radio, and I listen to my albums and my CDs, and just try and keep myself going. I think you have to say I’m not going to get down.”  “I want to try and write a book. We used to do that and then meet up every fortnight and talk about things and write things and we’ve been doing that on Zoom. We were doing it every week and it got to the stage actually where it was getting too much. So it's now down to every fortnight. So that's as a little social thing and keeping the mind active as well. Keep that going, yes.”  *And from Sky Arts on my big TV that has a few programmes a week. …compared with how often I might have been going to the theatre or cinema. I’ve been able to get performances there. It’s kept me happy.* |
|  | **Being outdoors and connecting with nature** | “I get up at 8 o’clock to do my lessons, I have a video lesson where I just sit waiting for it to end more or less because it’s not a lesson, it’s just a video on a screen that I have to view [?]. So, I do that in the morning, and then I eat lunch and I’m reading or doing nothing for an hour. And then at the end of school I can go out and either I go out for a short walk at lunch or I’ll go play football with my friend, and that’s more or less the only thing that’s enjoyable.”  *‘I did go out for the once-a-day exercise. I usually tried to do it after work just to give me that break to change my mindset from work mode to coming back to relaxing.’*  *Having plants grow within the house is an amazing way to keep track of time when it all seems so monotonous because it changes, it grows, and you are there. It’s a little project and I found that it’s so fantastic for me. I’ve really loved having my plants.*  “In the garden, I grow some fruit and veg, and I did a lot more gardening during lockdown, just to be outside but also, not socialising. That was a good stress relief.” | “I will go out, because it just calms me down, it makes me feel so much better, yes, just to get outside.”  “I sit in the garden and read a book or sit in the garden and meet a friend, but I am out in the fresh air. I’m enormously aided by the fact there are now three hedgehogs that come to my garden. So, I'm out there. I like to be out at dusk because it's cooler, and the air is so fresh. I take out the hedgehogs dinner and watch them while they come and get it and try not to tread on the young frogs that leap out of the pond. So, at least I get the fresh air.” |
| **Theme 6: Giving and receiving social and community support** | **Engaging with interest-based social groups** | “I have made a bunch of friends that are from online around my age from playing a game… so during the pandemic instead of chatting with my friends I’d be playing a game with them almost every day. Well, every day for a few hours chatting, having fun which was nice.”  “I'm quite lucky, with my bridge, I play international bridge, online. And that has been an absolute lifesaver for me, because it’s kept my brain ticking over a bit, and given me, just a challenge, so I'm very fortunate with that.”  *‘Since lockdown started, I’ve been involved in playing a lot more social board games online…and really built a wonderful community of people from all around the world who are really interesting people. …It’s a nice, different tangent.’* | “With the walking group, etc., it’s 55 to 65, 70 basically. Retired people. I get pleasure out of that,”  “Since lockdown started I’ve been involved in playing a lot more social board games online and this amazing community that I’ve entered and am talking with now. Over the months, I’ve started actually helping organise these games and run through the socials and advertise them and really built a wonderful community of people from all around the world who are really interesting people and are all connecting online.”  *Oh, and the other one I'm sort of involved, I was before lockdown, in a creative writing group… we’ve been doing that on Zoom. .... So that's as a little social thing and keeping the mind active as well. Keep that going, yes.* |
|  | **Volunteering and community participation** | “It was literally only one morning a week [that I volunteered at a food bank] but I always saw the same people and they were really nice. And again it was more like productivity, I just felt like I was being really helpful… I just genuinely feel like everything that I do at the moment is really worthwhile to someone, it’s always helping someone else in some way.” | “everybody just wants to do their bit, and my way of doing my bit, because I can’t do anything else, is participating in studies like yours.”  “I joined up doing surveys for YouGov and Ipsos and stuff like that, and expressing opinions and so on. And so I thought well I needed to do something to keep my mind busy. I wasn’t worried about my physical side of the body I was more concerned about my mind.”  *My way of doing my bit... is participating in studies like yours… because I can’t go out there and deliver meals to people… but what I can do is contribute to a body of knowledge, that hopefully will be helpful to people in the future.* |
