# Supplementary material for: A qualitative study of positive psychological experiences and helpful coping behaviours among young people and older adults in the UK during the COVID-19 pandemic
Source: PLoS One. 2023 Jan 23;18(1):e0279205. doi: 10.1371/journal.pone.0279205 (PMC9870142; doi:10.1371/journal.pone.0279205)
Supplement: Attachment — Submitted filename: ReviewerComments.Plos_One_final.docx [file pone.0279205.s005.docx]

# Editor Comments

| Editor Comments | Author Response |
| --- | --- |
| 1. Please ensure that your manuscript meets PLOS ONE's style requirements, including those for file naming. | Thank you - This has been actioned. |
| 2. It is in our understanding that the age of emancipation for research in the UK is 18 years of age. In the case of your study we have noted that for participants aged 13-15, they were asked to provide their assent and have a parent provide written informed consent. In your Methods section, please could you provide reference to a legal document indicating that participants above the age of 16 could provide informed consent independently for participation in research, and please could you also indicate whether the IRB approved the informed consent procedure described. | We appreciate the editor raising this important point. Adolescents in the UK aged 16-18 with a level of understanding sufficient to agree to participation in research were able to give their informed consent to participate in our research (see UCL ethics guidance [here](https://www.google.com/url?sa=t&rct=j&q=&esrc=s&source=web&cd=&cad=rja&uact=8&ved=2ahUKEwj-3vShiZD7AhWGd8AKHcB9AYcQFnoECBMQAQ&url=https%3A%2F%2Fethics.grad.ucl.ac.uk%2Fforms%2Fguidance1.pdf&usg=AOvVaw0I-9y32nVpVcy8LRhiZmWr)). There is no statute in England, Wales or Northern Ireland governing a child's right to consent to take part in research other than a Clinical Trial of an Investigational Medicinal Product (CTIMP), i.e. consent for non-CTIMPs. We followed [these guidelines from the UK Health Research Authority](https://www.hra.nhs.uk/planning-and-improving-research/policies-standards-legislation/research-involving-children/) regarding children’s’ involvement with clinical research as best practice.  The UCL Ethics Committee reviewed and approved all of our study procedures and this has been articulated in the Methods section (page 6-7). |
| 3. Thank you for stating the following financial disclosure:     "This research was supported by Nuffield Foundation [WEL/FR-000022583], but the views expressed are those of the authors and not necessarily the Foundation. The study was also supported by the MARCH Mental Health Network funded by the Cross-Disciplinary Mental Health Network Plus initiative supported by UK Research and Innovation [ES/S002588/1], and by the Wellcome Trust [221400/Z/20/Z]. "  Please state what role the funders took in the study.  If the funders had no role, please state: "The funders had no role in study design, data collection and analysis, decision to publish, or preparation of the manuscript."  If this statement is not correct you must amend it as needed.  Please include this amended Role of Funder statement in your cover letter; we will change the online submission form on your behalf. | We confirm that this statement is correct and has now been updated and added to our cover letter. |
| 4. Thank you for stating the following in the Acknowledgments Section of your manuscript:      "This research was supported by Nuffield Foundation [WEL/FR-000022583], but the views expressed are those of the authors and not necessarily the Foundation. The study was also supported by the MARCH Mental Health Network funded by the Cross-Disciplinary Mental Health Network Plus initiative supported by UK Research and Innovation [ES/S002588/1], and by the Wellcome Trust [221400/Z/20/Z]. LO thanks Chevening Scholarships, the UK government’s global scholarship programme, funded by the Foreign, Commonwealth and Development Office (FCDO) and partner organisations University College London and Jeffrey Cheah Foundation for support towards pursuit of a master’s degree that facilitated undertaking of this study. The researchers are grateful for the support of AgeUK, Alzheimer’s Society, Healthwise Wales and British Youth Music Theatre during recruitment. We are grateful to Anna Roberts, Joanna Dawes, Louise Baxter, Sara Esser, Rana Conway and Tom May for their help with conducting interviews. The authors extend thanks to study  participants for their valuable contributions."  We note that you have provided funding information that is not currently declared in your Funding Statement. However, funding information should not appear in the Acknowledgments section or other areas of your manuscript. We will only publish funding information present in the Funding Statement section of the online submission form.  Please remove any funding-related text from the manuscript and let us know how you would like to update your Funding Statement. Currently, your Funding Statement reads as follows:   "This research was supported by Nuffield Foundation [WEL/FR-000022583], but the views expressed are those of the authors and not necessarily the Foundation. The study was also supported by the MARCH Mental Health Network funded by the Cross-Disciplinary Mental Health Network Plus initiative supported by UK Research and Innovation [ES/S002588/1], and by the Wellcome Trust [221400/Z/20/Z]. "  Please include your amended statements within your cover letter; we will change the online submission form on your behalf. | Thank you for pointing this out. Funding related information has now been removed from the acknowledgements section. The correct funding statement has been added to the cover letter. Reference to funding has been removed from the manuscript as requested. |
| 5. In your Data Availability statement, you have not specified where the minimal data set underlying the results described in your manuscript can be found. PLOS defines a study's minimal data set as the underlying data used to reach the conclusions drawn in the manuscript and any additional data required to replicate the reported study findings in their entirety. All PLOS journals require that the minimal data set be made fully available. For more information about our data policy, please see http://journals.plos.org/plosone/s/data-availability.  "Upon re-submitting your revised manuscript, please upload your study’s minimal underlying data set as either Supporting Information files or to a stable, public repository and include the relevant URLs, DOIs, or accession numbers within your revised cover letter. For a list of acceptable repositories, please see http://journals.plos.org/plosone/s/data-availability#loc-recommended-repositories. Any potentially identifying patient information must be fully anonymized.  Important: If there are ethical or legal restrictions to sharing your data publicly, please explain these restrictions in detail. Please see our guidelines for more information on what we consider unacceptable restrictions to publicly sharing data: http://journals.plos.org/plosone/s/data-availability#loc-unacceptable-data-access-restrictions. Note that it is not acceptable for the authors to be the sole named individuals responsible for ensuring data access.  We will update your Data Availability statement to reflect the information you provide in your cover letter. | We have now added a minimal dataset as requested with anonymised summaries that helped inform the scientific conclusions drawn on in the manuscript. We are unable to share the full dataset due to the possibility that this could compromise participant anonymity. |
| 6. Please include your full ethics statement in the ‘Methods’ section of your manuscript file. In your statement, please include the full name of the IRB or ethics committee who approved or waived your study, as well as whether or not you obtained informed written or verbal consent. If consent was waived for your study, please include this information in your statement as well. | Thank you for this suggestion. This information was included in the Methods section; however, we have now included a subsection on Ethics so that this is clearer. |
| 7. Please include captions for your Supporting Information files at the end of your manuscript, and update any in-text citations to match accordingly. Please see our Supporting Information guidelines for more information: http://journals.plos.org/plosone/s/supporting-information. | Many thanks for pointing this out. This has now been added to the end of the manuscript on page 35 |
| 8. Please review your reference list to ensure that it is complete and correct. If you have cited papers that have been retracted, please include the rationale for doing so in the manuscript text, or remove these references and replace them with relevant current references. Any changes to the reference list should be mentioned in the rebuttal letter that accompanies your revised manuscript. If you need to cite a retracted article, indicate the article’s retracted status in the References list and also include a citation and full reference for the retraction notice. | The authors have now re-checked each individual reference source as instructed and confirm that the reference list is correct. |

# Reviewer Comments: Reviewer 1

| Section/Comment | Author Response |
| --- | --- |
| Overall | |
| This is an excellent manuscript which coherently details the secondary qualitative analysis of interviews conducted with young and older adults in the UK during the COVID-19 pandemic.  The study aimed to assess the experiences of young and older adults through a 'positive psychology' lens by asking the question “what were the positive psychological experiences or coping behaviours that protected mental health and well-being of young people and older adults living in the UK during the COVID-19 pandemic?”  I believe the qualitative methods that are described in this paper to be methodologically rigorous and well explained. The paper follows the SRQR guidelines for reporting qualitative research.  The results are well presented, including tables which compare and constant younger and older adult experiences.  The discussion provided a in depth review of how the findings of this study can be situated within existing literature. | Many thanks to the reviewer for the positive comments on our manuscript. |

# Reviewer 2

| Section/Comment | Author Response |
| --- | --- |
| Overall | |
| I want to thank you for the opportunity to review this interesting study describing the positive psychological experiences and coping behaviours of young people and older adults living in the UK during the COVID-19 pandemic. In my humble opinion, it offers interesting results that the scientific community and society in general can benefit from. I believe that the topic is original, the manuscript is very well written, the analyses seem straightforward, and the results can make a significant contribution to relevant literature. The discussion of the results holds significant suggestions that are highly appreciated. | Many thanks to the reviewer for the positive comments on our manuscript. |

# Reviewer 3

| Section/Comment | Author Response |
| --- | --- |
| Overall | |
| The paper explored positive psychological experiences and coping behaviours that protected mental health and well-being in young people (aged 13-24) and older adults (aged 70+) living in the UK during the COVID-19 pandemic. The article adequately fits in the profile of Plos One. Nevertheless, I have some minor concerns. | Many thanks to the reviewer for their helpful suggestions on our manuscript. We have addressed these minor revisions in the attached file and summarise these below. |
| In general, it is a useful but a repetitive paper that should be shortened in some parts to facilitate the reading and the comprehension. | Thank you for this feedback. We have shortened some sections that might be seen as repetitive. |
| Methods | |
| 1. More information should be given about the interviews, where they took place, how. | We thank the reviewer for their suggestions on how we might improve the Methods section of the manuscript. We have re-arranged information about the interviews to make this clearer (page 7). |
| 2. How exactly the authors assess physical and mental health conditions? They suffer from depression/anxiety during the study/the lockdown or before? | Physical and mental health data were self-reported, collected via a demographics questionnaire. We have added further detail in the Methods section to make this clearer (page 7). Although some participants spontaneously discussed a new depression or anxiety diagnosis during their interview, we did not routinely ask all participants to specify when their health condition was diagnosed. |
| 3. All participants were informed about the confidentiality of the information collected? If yes, this sentence should be added | Yes – participants were informed about the confidentiality of information collected. Further text has now been added on page 8. |
| 4. Why the second researcher only revise 3 and not all the transcripts in order to be more objective? | The dataset is considered extremely large for a qualitative interview study and to have a second researcher review all transcripts would not have been feasible. We did utilize various data triangulation strategies, including attainment of team consensus on themes and inclusion of verbatim quotes, to minimize influence of individual-level researcher biases and thereby enhance trustworthiness of findings (Nowell et al., 2017). |
| 5. There is a typo in table 1 footnote: “Carribean” instead of “Caribbean” | Thank you very much for pointing out this error, which has now been amended. |
| 6. Clap for Carers” (a social movement in appreciation of people working for the UK’s National Health Service). This explanation should appear in the column “overall” | Thank you for this suggestion, which has now been reflected in Table 2 (page 12). |
| Results | |
| 7. Why Table 1 is divided in several age groups? It is not explained before, and neither it is used in results or discussion section | Many thanks for raising this. This was done to provide context for interpretation of demographic data eg status of employment and living situation, as well as provide context for interpretation of our findings with regards to different life stages. |
